# Strategies to Prevent Cardiac Implantable Electronic Device Infection

**DOI:** 10.19102/icrm.2020.110102

**Published:** 2020-01-15

**Authors:** Tarek Barbar, Rohan Patel, George Thomas, Jim W. Cheung

**Affiliations:** ^1^Division of Cardiology, Department of Medicine, Weill Cornell Medicine–New York Presbyterian Hospital, New York, NY, USA

**Keywords:** Cardiac implantable electronic devices, implantable cardioverter-defibrillators, infection, pacemakers

## Abstract

The association between the risk of mortality and cardiovascular implantable electronic device (CIED) infections has been well-established in the literature. As CIED implantations have increased in frequency in the past few decades, the incidence of CIED-related infections has also risen. Given the morbidity, mortality, and health-care costs associated with CIED infections, the prevention of device-related infection is a critical goal. Risk factors for developing CIED infections can be categorized as patient-, procedure-, or device-related. Numerous studies have highlighted different strategies for preventing CIED-related infections, which include patient optimization, device selection, and periprocedural preparation and treatment. Nonetheless, as the comorbidity burden of patients undergoing CIED implantation continues to increase, significant challenges in the successful elimination of CIED-related infections remain. This review provides a comprehensive overview of available evidence-based approaches and strategies to reduce the risk of CIED infections.

## Introduction

During the past several decades, cardiovascular implantable electronic devices (CIEDs) such as implantable cardioverter-defibrillators (ICDs) and cardiac resynchronization therapy (CRT) systems have led to significant reductions in cardiac morbidity and mortality.^1–4^ As the indications for device implantation have expanded, the use of CIEDs has significantly increased. Between 1997 and 2004, the rates of ICD and pacemaker (PPM) implantations in the United States increased by 60% and 19%, respectively.^5^ A further dramatic increase in implantation rates occurred between 2004 and 2008, resulting in overall increases in ICD and PPM implantation rates of 500% and 45%, respectively, over 16 years (1993–2008). Overall, CIED implantations in the United States increased by more than 95% during that time period.^6^

Alongside the rise in CIED implantation, the frequency of CIEDs infections has also increased; for example, from 1993 to 2008, the incidence of CIED infections rose by more than 200%.^6^ The absolute rate of CIED infections grew from almost 1.5% in 2004 to at least 2.4% in 2008. At this time, the growth in the CIED infection rate appears to have outpaced the increase in the rate of device implantations.^7^ This may be explained in part by the existence of more comorbidities among candidates for device placement and greater device complexity. Furthermore, the increased overall survival rate among CIED recipients can also facilitate a higher risk of developing a CIED-associated infection over time.^8^ CIED infections can vary in extent from localized pocket infections to systemic infections with lead and valvular involvement.^9^ According to the National Hospital Discharge Survey, the risk of in-hospital mortality was increased more than twofold among patients with CIED infections.^10^ In a study of 200,219 Medicare patients, those with CIED infections had a twofold higher mortality rate at one year, which remained elevated even at three years, after device implant.^11^ In addition to provoking heightened morbidity and mortality rates, CIED infections can lead to significant health-care expenditures. It has been reported that the average cost associated with the treatment of a single CIED infection is $146,000.^6^ Given the significant public health burden of CIED infections, updated guidelines for their prevention have been developed.^12^ These strategies include the proper selection of patients eligible for CIED placement, the optimization of sterile device implantation techniques, and the use of perioperative antibiotics and antibiotic-impregnated mesh envelopes.^13^ In this report, we present an overview of the different approaches and strategies available for reducing the risk of CIED infections **(Table 1)**.

## Pathogenesis of cardiac implantable electronic device infections

The most common mechanism for developing a CIED infection is local contamination of the device itself, its leads, or the pocket during implantation.^14,15^ Inoculation can occur with the presence of skin breaks that introduce contaminants into the pocket. Almost two-thirds of CIED infectious complications are pocket infections.^16^ Given that pocket sources are the dominant mechanism of CIED infections, many CIED infections are the result of Gram-positive organisms, with the majority of these being due to coagulase-negative staphylococci.^16,17^ One of the most important virulence factors for some of these pathogens is biofilm formation.^15^ Several strategies to target biofilm formation have been suggested, including the use of bioelectric and bioacoustic therapy. In in vitro models, the application of low electrical current in combination with antimicrobial agents have been shown to enhance the killing of biofilm-associated bacteria.^18^ In an animal model, ultrasound application combined with gentamicin greatly reduced bacterial viability.^18^ These strategies have yet to be fully investigated in human studies.

Another mechanism of CIED infection is device seeding via hematogenous spread from a focal infection located elsewhere in the body.^15^ This can often be associated with infective endocarditis. In a retrospective cohort study by Uslan et al., more than one-third of patients with CIEDs and *Staphylococcus aureus* bacteremia had CIED infections.^19^ Of the patients with CIED infections, half had evidence of CIED-related endocarditis. The management of CIED-related infections includes the complete removal of the device with its accessory hardware, as the use of systemic antibiotics alone will not suffice.^15^ Therefore, the prevention of CIED infections is essential given the risks associated with the treatment of systemic infections.

## Risk factors for cardiac implantable electronic device infection

Identifying risk factors associated with CIED complications is an integral part of infection prevention. CIED infection risk factors can be categorized as either patient-, procedure-, or device-related.^20^ Several patient-level risk factors have been associated with CIED infections. Previous studies have shown an association between the male gender and an increased risk of CIED infections, although the underlying mechanism of this link is unclear.^21–23^ Notably, despite the higher rates of CIED infections among men, cases of such among women are more likely to be associated with mortality.^24^ The causes of this difference in mortality from CIED infections vary and include gender-related differences in the recognition of CIED infections and physiologic responses to sepsis. Data on age as a CIED infection risk factor have yielded conflicting results, with some studies suggesting increased risks of CIED infections among older patients^25,26^ and others identifying higher rates of CIED infections among younger patients.^23^ Confounding age-associated factors such as comorbidities can limit the conclusions that can be made about age as an independent predictor of CIED infection. Renal failure, diabetes mellitus, and chronic obstructive pulmonary disease (COPD) have all been associated with CIED infections.^20^ Irrespective of a concurrent need for hemodialysis, renal failure is a strong risk factor for CIED infections, especially in patients with associated diabetes.^27,28^ Diabetes mellitus and COPD were each found to be independent risk factors of CIED infectious complications with odds ratios (ORs) of 3.5 and 3, respectively.^29,30^

The presence of fever or leukocytosis prior to CIED implantation can also be a risk factor for device infections. In the Prospective Evaluation of Pacemaker Lead Endocarditis (PEOPLE) study, a large prospective survey of more than 6,000 patients, the presence of fever within 24 hours prior to CIED implantation was positively correlated with a higher risk of developing device-related infections (adjusted OR: 4.8).^31^ However, the correlation between isolated leukocytosis and CIED infection is less clear. A recent study showed no significant association existed between device infections and preoperative isolated leukocytosis in the absence of other infectious markers such as bacteremia, fever, or physical examination results suggesting an ongoing infectious process.^32^

The use of certain medications including anticoagulants and corticosteroids is significantly associated with a higher rate of CIED-related infectious complications. Cengiz et al. showed that at least 15% more infectious events were noted among patients on anticoagulants.^25^ In the setting of corticosteroid use for more than one month, the absolute increase in the incidence of CIED infectious complications was 5%.^25^ The presence of a permanent central venous catheter has also been shown to be an independent predictor of CIED infections.^25,33^ Finally, patients with a prior history of device revisions, generator replacements, and prior infections have higher risks for contracting a CIED infection.^30,33,34^ In particular, the incidence of infection in conjunction with replacement procedures was at least twofold higher than that of infection presenting in relation to de novo implantations.^35^

Procedure-related factors have also been shown to lead to a significantly increased risk for infections. The lack of preoperative antibiotic prophylaxis has been identified as an independent risk factor for CIED infections. After over 30 years of general consensus on the importance of preoperative antibiotics for the prevention of CIED infections, it was not until 2009 that prospective randomized clinical trial data demonstrated significantly lower infectious complications in patients who received preoperative cefazolin than those who did not.^36^ Postoperative pocket hematomas have been positively correlated with CIED infections as well. In one study, the presence of a postoperative hematoma was associated with an almost sevenfold increase in the rate of CIED infection.^37^ This finding supports the observed correlation between anticoagulant use and increased CIED infection risk. Multiple studies have revealed that the need for temporary pacing, device revisions, and increased procedural duration significantly increase the rate of CIED infections.^31,38,39^ Reinterventions have been found to increase the rate of CIED infections. One prospective cohort study of 316 patients identified a nearly eightfold increased risk of infection as associated with device reintervention procedures.^38^ Furthermore, there is a correlation between the timing of reintervention and the risk of CIED infections. In one study, early reinterventions, defined as repeat procedures occurring during index admission prior to discharge, were associated with a more than 15-fold increased risk of CIED infection.^31^ Low levels of implant physician experience have also been associated with increased risks of CIED infection. In an analysis of Medicare data, the risk of 90-day infections after ICD implant was almost 2.5-fold higher among implanters who performed only one to 10 implants per year than among those who performed 29 or more implants per year.^40^ Whether this association is due to longer procedure durations or suboptimal patient preparation prior to implantation among lower-volume operators is unclear. In a prospective study, longer procedures (an average of 85 minutes versus almost 60 minutes) were independently associated with infectious complications.^38^

Further, the complexity and location of the device implanted may be associated with different risks of infectious complications. Studies have shown that dual-chamber device implantations carry a higher risk of infection than single-chamber device implantations.^33,41^ Further, CRT defibrillators have been associated with a higher risk of infection as compared with ICDs.^42^ Overall, the number of leads present can be an independent risk factor for device-related infections. In one case–control study, patients who had more than two pacing leads were at a higher risk of infection than those with two pacing leads only (OR: 5.41).^33^ The presence of abandoned leads was not associated with an increased risk of infection. It is unclear as to whether infection risk associated with a greater number of device leads is related to the presence of additional hardware itself or is just a reflection of longer procedure times during lead implantation. Finally, retrospective data suggest that devices implanted abdominally have a higher CIED infection rate than pectorally implanted ones.^43,44^ Overall, it should be noted that higher rates of CIED infection among patients with more complex devices or abdominal implants may be attributable in part to a higher comorbidity burden rather than just device-related factors.

## Prevention of cardiac implantable electronic device infection

### Patient selection and optimization

The prevention of CIED infections requires a multipronged approach that addresses all of the patient-, procedural-, and device-related risk factors. Prior to performing a CIED procedure, appropriate patient selection and optimization is a critical first step. Given that a considerable proportion of patients receiving CIEDs are older and have a significant comorbidity burden, optimizing the management of coexisting diseases will help reduce the risk of device-related infectious complications.^20^ The presence of fever should prompt the consideration of procedure postponement to permit diagnosis and treatment to occur for potential preexisting infections. Any indwelling central venous catheters that are not absolutely required for further patient treatment should be removed prior to CIED implantation.

### Device selection

As discussed earlier, the risk of infection can vary based on the type of device implanted or the number of leads placed. Newer devices such as leadless PPMs and subcutaneous ICDs (S-ICDs) have emerged as potential alternatives to transvenous devices and may theoretically have features that can reduce CIED-related infections. However, whether or not these devices actually significantly reduce infection rates remains to be established. In fact, some research has shown that the overall infection rate of S-ICDs is not significantly different from that of transvenous ICDs.^45^ However, theoretically, because S-ICDs do not contain any intravascular components, the risk of systemic infection should be low. Nonetheless, systemic involvement of S-ICD infections has been previously reported.^46,47^ The management of subcutaneous ICD infections may to be easier given that transvenous lead extraction is not required with subcutaneous ICD removal.^17,46,48^ Ultimately, the completion of the Prospective, Randomized Comparison of Subcutaneous and Transvenous ICD Therapy (PRAETORIAN) clinical trial will better clarify whether or not the implantation of S-ICDs can lead to reduced infection-related adverse events relative to the implantation of transvenous ICDs.^49^ The impact of leadless pacing technology on reducing the long-term risks of CIED infection remains to be seen. Theoretically, the absence of a pacemaker pocket and transvenous lead may reduce the risk of primary device infection associated with leadless pacemakers.^50^ However, hematogenous seeding of the device by a remote-site infection may still be possible.

### Preoperative preparation and intraoperative considerations

Implementing optimal procedural-related strategies and proper aseptic techniques constitute some of the simplest yet most effective ways to decrease the infection risk during device implantation. Patients should wear masks and operating staff should wear gowns and masks.^51^ Since CIED implantation is a surgical procedure, adherence to regulations for general surgical procedures to reduce wound infections should be adopted. For surgical site preparation, Seropian and Reynolds found that hair removal with shaving using a razor prior to the procedure may increase the risk of wound infections.^52^ Current recommendations stipulate that preoperative hair removal should not be performed unless it interferes with the surgical site.^51^ If hair removal is necessary, then the use of electronic clippers rather than shaving is preferred. Following clipping, skin-site preparation should continue with a topical cleanser. The optimal choice of topical antiseptic is debatable. In a randomized clinical trial of 849 patients, a chlorhexidine–alcohol antiseptic cleanser was associated with a 41% relative reduction in the rate of surgical-site infections when compared with a povidone–iodine scrub.^53^ However, a single-center cohort study of patients receiving CIEDs failed to observe a difference in infection rates between patients using either topical antiseptic.^54^ Iodine-impregnated drapes can also be considered for use, although there are no data in the CIED literature to suggest that their inclusion reduces the risk of infection.

Finally, maintaining an appropriate environment in the operating room is important in further reducing the risk of procedure-related CIED infections. This includes the presence of a proper ventilation system with positive pressure in the operating room, the optimization of air quality with filtered air and frequent air exchanges, and restricting the number of personnel present in the room during the procedure as well as confirmation that all involved individuals are wearing the required protective equipment.^55^

### Postoperative hematoma prevention

Given the association between postprocedural hematoma formation and the increased risk of CIED infection,^37^ strategies to prevent postoperative hematomas have been recommended, which include the placement of pressure dressings, the scrupulous use of electrocauterization, and the administration of hemostatic agents.^56^ Some of the hemostatic agents studied include microporous polysaccharide hemostatic (MPH) powder (Arista; Bard, Warwick, RI, USA), oxidized regenerated cellulose (Surgicel Fibrillar Hemostat; Johnson & Johnson, New Brunswick, NJ, USA), local tranexemic acid (TXA), and topical thrombin. The use of MPH was shown to decrease the rate of pocket hematoma formation by 70% in one study.^57^ Elsewhere, the application of oxidized regenerated cellulose was studied in 42 patients receiving CIEDs while remaining on either warfarin or dual antiplatelet therapy (DAPT).^58^ This investigation reported no case of pocket hematoma formation with six months of follow-up. A retrospective analysis was conducted to assess the effects of TXA on preventing hematoma formation post-CIED implantation.^59^ The study included 135 patients who were either on warfarin or DAPT or warfarin plus DAPT. The study revealed a significant decrease in pocket hematoma formation in patients receiving TXA (7.7% versus 26.5%). In contrast, a collagen and thrombin blend (D-Stat; Teleflex, Morrisville, NC, USA) failed to promote a significant reduction in hematoma formation.^60^

Avoiding heparin products perioperatively has also been suggested to reduce the risk of hematoma formation.^61^ In the Bridge or Continue Coumadin for Device Surgery Randomized Controlled Trial (BRUISE CONTROL) study, the risk of hematoma formation was assessed in patients continued on uninterrupted warfarin versus heparin bridging periprocedurally. There was a significantly higher risk of pocket hematoma formation in patients who received heparin bridging (16% versus 3.5%), suggesting uninterrupted warfarin therapy to be a safer option. BRUISE CONTROL-2 examined the risk of hematoma formation in patients on direct oral anticoagulants (DOACs).^62^ Patients were randomized to either uninterrupted or interrupted DOAC therapy periprocedurally, and no significant difference in hematoma formation was found between the two groups.

For patients with large pocket hematomas in whom there was a high risk of wound dehiscence, pocket hematoma evacuation should be considered. However, early reintervention procedures can also increase the risk of device-related infectious complications.^31,38^ Therefore, the decision to proceed with hematoma evacuation requires a careful weighing of the risks and benefits of early reintervention.

### Prophylactic antibiotics

The role of systemic prophylactic antibiotic administration in reducing CIED infections has been studied extensively **(Table 2)**. The Centers for Medicare and Medicaid Services, in collaboration with the Centers for Disease Control and Prevention, introduced guidelines recommending the prophylactic perioperative administration of antimicrobial agents.^63^ The American Heart Association and Heart Rhythm Society have recommended the preoperative administration of prophylactic antibiotics as such has been associated with a lower rate of CIED infections.^56,64^ The use of intravenous cefazolin has been found to significantly decrease the incidence of CIED infections when compared with placebo (0.63% versus 3.28%).^36^ The Prevention of Arrhythmia Device Infection Trial (PADIT) examined 19,603 high-risk patients undergoing device procedures who were randomized to an incremental perioperative antibiotic approach or a standard preoperative antibiotic approach.^65^ The incremental approach consisted of standard preoperative cefazolin dosing followed by an intraoperative bacitracin wash and postoperative oral cephalexin for two days. The study showed no statistically significant difference in rates of CIED infections between patients assigned to the incremental approach and the standard approach, respectively. Notably, the overall rate of infection was low at around 1%, which establishes a reasonable hospital benchmark for tracking CIED procedure outcomes for quality improvement.

### Antibiotic-impregnated envelopes

Aside from systemic antibiotic approaches to reduced CIED infections, a localized antibiotic approach using an antibiotic-impregnated envelope has been evaluated in a large randomized clinical trial of more than 6,900 patients considered at high risk for infection **(Table 2)**. The Worldwide Randomized Antibiotic Envelope Infection Prevention Trial (WRAP-IT) examined the TYRX envelope (Medtronic, Minneapolis, MN, USA), which consists of a mesh coated with an absorbable polymer mixed with minocycline and rifampin, which are eluted into the local tissue for at least seven days.^66^ Investigators reported that, at 12 months, a significantly lower rate of CIED infections was observed among patients who received the envelope in comparison with those who did not (0.7% versus 1.2%, respectively). Further, there was no increased complication rate associated with the use of the envelope. Given the low overall rate of CIED infections, the number of envelopes needed to prevent a single CIED infection at one year would be 200 based on the absolute risk reduction of infection with the use of said envelopes. Given the high costs of readmissions and procedures associated with CIED infections, a cost–benefit analysis may show the use of an antibiotic-impregnated envelope to be cost-effective, especially when used among patients at a high risk of infection.

### Remaining challenges and future directions

Despite all of the efforts made to prevent the onset of infectious complications of CIED implantations, we still face challenges that limit our ability to fully eliminate the risk of such. As patients live longer with CIEDs and the comorbidity burden of individuals undergoing CIED procedures continues to increase, the continued evolution of technology and best practices to reduce CIED infection will be paramount. Improvements in leadless pacing technology that incorporate dual-chamber and CRT pacing functionalities could contribute to a decrease in CIED pocket infections, although this will require extensive future study.

## Conclusion

CIED infections are strongly associated with increased morbidity and mortality rates and add substantial financial burden to our health-care system. A multipronged strategy to prevent CIED infection is required. The proper selection and optimization of patients before performing a CIED implant is mandatory. Basic preparation of the operating theater and the patient, which includes proper skin preparation and the use of perioperative antibiotics, is essential. Efforts to reduce hematoma formation and lead complications requiring early reintervention can reduce the risk of CIED infection. The use of an antibiotic-impregnated mesh envelope during implantation in high-risk individuals may also help to reduce infections. Despite the evolution of these CIED infection prevention strategies, significant challenges remain.

## Figures and Tables

**Table 1:** Summary of Measures to Reduce CIED Infections

Category	Intervention
Patient selection	• Optimization of comorbidities prior to implantation • Delaying implantation in the setting of fever or leukocytosis with other infectious markers (eg, bacteremia, physical examination consistent with an infectious process) • Removal of central venous catheters prior to implantation
Device selection	• Consideration of leadless pacemakers or subcutaneous ICDs if appropriate
Provider preparation	• Use of appropriate gowns and masks
Surgical site preparation	• Electronic hair-clipping instead of shaving • Chlorhexidine–alcohol antiseptic cleanse
Operating theater conditions	• Proper ventilation system • Air-quality optimization • Restriction of number of personnel • Temperature control
Hematoma prevention	• Use of pressure dressings • Use of electrocautery • Avoidance of heparin products
Other postoperative considerations	• Avoid early reintervention unless necessary • Pocket evacuation in the case of higher dehiscence risk
Prophylactic antibiotics	• Preoperative use of intravenous cefazolin
Antibiotic-impregnated envelopes	• Consideration of use of minocycline-/rifampin-impregnated mesh envelope in high-risk patients

CIED: cardiovascular implantable electronic device; ICD: implantable cardioverter defibrillator.

**Table 2:** Summary of Randomized Clinical Trials on Antibiotic Strategies to Reduce CIED infections

Trial/Study Name	Number of Participants (n)	Study Population	Study Design	Follow-up Duration	Results
Heart Institute University of São Paulo^36^	649	PPM and ICD implantation or generator replacement (high-risk patients excluded)	Cefazolin vs. placebo preoperative prophylaxis	6 months (study interrupted early)	Decreased CIED infection rate with cefazolin (0.64%) vs. placebo (3.28%) (p = 0.016)
PADIT^65^	19,603	PPM, ICD, and CRT-ICD implantation (high-risk patients included)	Preoperative prophylactic cefazolin or vancomycin (in PCN-allergic patients) vs. incremental preoperative, intraoperative, and postoperative antibiotics (eg, cefazolin/vancomycin, bacitracin wash, cephalexin/cephadroxil)	6 months	No significant difference in CIED infection rate (1.03% in conventional arm and 0.78% in incremental arm) (p = 0.10)
WRAP-IT^66^	6,983	High-risk CIED implantation (eg, replacement, upgrade, revision, or CRT procedures)	Antibiotic-impregnated mesh envelope vs. control	12 months	Decreased CIED infection rate with envelope (0.7%) vs. control (1.2%) (p = 0.04)

CIED: cardiac implantable electronic device; CRT: cardiac resynchronization therapy; ICD: implantable cardioverter-defibrillator; PPM: permanent pacemaker.

## References

[1] Bardy GH, Lee KL, Mark DB (2005). Amiodarone or an implantable cardioverter-defibrillator for congestive heart failure.. N Engl J Med..

[2] Linde C, Abraham WT, Gold MR (2008). Randomized trial of cardiac resynchronization in mildly symptomatic heart failure patients and in asymptomatic patients with left ventricular dysfunction and previous heart failure symptoms.. J Am Coll Cardiol..

[3] Moss AJ, Hall WJ, Cannom DS (2009). Cardiac-resynchronization therapy for the prevention of heart-failure events.. N Engl J Med..

[4] Cazeau S, Leclercq C, Lavergne T (2001). Effects of multisite biventricular pacing in patients with heart failure and intraventricular conduction delay.. N Engl J Med..

[5] Zhan C, Baine WB, Sedrakyan A, Steiner C (2008). Cardiac device implantation in the United States from 1997 through 2004: a population-based analysis.. J Gen Intern Med..

[6] Greenspon AJ, Patel JD, Lau E (2011). 16-year trends in the infection burden for pacemakers and implantable cardioverter-defibrillators in the United States 1993 to 2008.. J Am Coll Cardiol..

[7] Voigt A, Shalaby A, Saba S (2010). Continued rise in rates of cardiovascular implantable electronic device infections in the United States: temporal trends and causative insights.. Pacing Clin Electrophysiol..

[8] Cabell CH, Heidenreich PA, Chu VH (2004). Increasing rates of cardiac device infections among Medicare beneficiaries: 1990–1999. Am Heart J..

[9] Baddour LM, Cha YM, Wilson WR (2012). Clinical practice. Infections of cardiovascular implantable electronic devices.. N Engl J Med..

[10] Voigt A, Shalaby A, Saba S (2006). Rising rates of cardiac rhythm management device infections in the United States: 1996 through 2003.. J Am Coll Cardiol..

[11] Rizwan Sohail M, Henrikson CA, Jo Braid-Forbes M, Forbes KF, Lerner DJ (2015). Increased long-term mortality in patients with cardiovascular implantable electronic device infections.. Pacing Clin Electrophysiol..

[12] Epstein AE, DiMarco JP, Ellenbogen KA (2008). ACC/AHA/HRS 2008 Guidelines for Device-Based Therapy of Cardiac Rhythm Abnormalities: a report of the American College of Cardiology/American Heart Association Task Force on Practice Guidelines (Writing Committee to Revise the ACC/AHA/NASPE 2002 Guideline Update for Implantation of Cardiac Pacemakers and Antiarrhythmia Devices) developed in collaboration with the American Association for Thoracic Surgery and Society of Thoracic Surgeons.. J Am Coll Cardiol..

[13] Rohacek M, Baddour LM (2015). Cardiovascular implantable electronic device infections: associated risk factors and prevention.. Swiss Med Wkly..

[14] Da Costa A, Lelievre H, Kirkorian G (1998). Role of the preaxillary flora in pacemaker infections: a prospective study.. Circulation..

[15] Nagpal A, Baddour LM, Sohail MR (2012). Microbiology and pathogenesis of cardiovascular implantable electronic device infections.. Circ Arrhythm Electrophysiol..

[16] Sohail MR, Uslan DZ, Khan AH (2007). Management and outcome of permanent pacemaker and implantable cardioverter-defibrillator infections.. J Am Coll Cardiol..

[17] Margey R, McCann H, Blake G (2010). Contemporary management of and outcomes from cardiac device related infections.. Europace..

[18] Patel R (2005). Biofilms and antimicrobial resistance.. Clin Orthop Relat Res..

[19] Uslan DZ, Dowsley TF, Sohail MR (2010). Cardiovascular implantable electronic device infection in patients with Staphylococcus aureus bacteremia.. Pacing Clin Electrophysiol..

[20] Polyzos KA, Konstantelias AA, Falagas ME (2015). Risk factors for cardiac implantable electronic device infection: a systematic review and meta-analysis.. Europace..

[21] Catanchin A, Murdock CJ, Athan E (2007). Pacemaker infections: a 10-year experience.. Heart Lung Circ..

[22] Johansen JB, Jorgensen OD, Moller M, Arnsbo P, Mortensen PT, Nielsen JC (2011). Infection after pacemaker implantation: infection rates and risk factors associated with infection in a population-based cohort study of 46299 consecutive patients.. Eur Heart J..

[23] Lin YS, Hung SP, Chen PR (2014). Risk factors influencing complications of cardiac implantable electronic device implantation: infection, pneumothorax and heart perforation: a nationwide population-based cohort study.. Medicine (Baltimore)..

[24] Sohail MR, Henrikson CA, Braid-Forbes MJ, Forbes KF, Lerner DJ (2013). Comparison of mortality in women versus men with infections involving cardiovascular implantable electronic device.. Am J Cardiol..

[25] Cengiz M, Okutucu S, Ascioglu S (2010). Permanent pacemaker and implantable cardioverter defibrillator infections: seven years of diagnostic and therapeutic experience of a single center.. Clin Cardiol..

[26] Tan EM, DeSimone DC, Sohail MR (2017). Outcomes in patients with cardiovascular implantable electronic device infection managed with chronic antibiotic suppression.. Clin Infect Dis..

[27] Bloom H, Heeke B, Leon A (2006). Renal insufficiency and the risk of infection from pacemaker or defibrillator surgery.. Pacing Clin Electrophysiol..

[28] Tompkins C, McLean R, Cheng A (2011). End-stage renal disease predicts complications in pacemaker and ICD implants.. J Cardiovasc Electrophysiol..

[29] Herce B, Nazeyrollas P, Lesaffre F (2013). Risk factors for infection of implantable cardiac devices: data from a registry of 2496 patients.. Europace..

[30] Sohail MR, Hussain S, Le KY (2011). Risk factors associated with early- versus late-onset implantable cardioverter-defibrillator infections.. J Interv Card Electrophysiol..

[31] Klug D, Balde M, Pavin D (2007). Risk factors related to infections of implanted pacemakers and cardioverter-defibrillators: results of a large prospective study.. Circulation..

[32] Kumar DS, Tompkins CM, Veenhuyzen GD, Henrikson CA (2018). Significance of leukocytosis prior to cardiac device implantation.. Pacing Clin Electrophysiol..

[33] Sohail MR, Uslan DZ, Khan AH (2007). Risk factor analysis of permanent pacemaker infection.. Clin Infect Dis..

[34] Ann HW, Ahn JY, Jeon YD (2015). Incidence of and risk factors for infectious complications in patients with cardiac device implantation.. Int J Infect Dis..

[35] Arana-Rueda E, Pedrote A, Frutos-Lopez M (2017). Repeated procedures at the generator pocket are a determinant of implantable cardioverter-defibrillator infection.. Clin Cardiol..

[36] de Oliveira JC, Martinelli M, Nishioka SA (2009). Efficacy of antibiotic prophylaxis before the implantation of pacemakers and cardioverter-defibrillators: results of a large, prospective, randomized, double-blinded, placebo-controlled trial.. Circ Arrhythm Electrophysiol..

[37] Sadeghi H, Alizadehdiz A, Fazelifar A, Emkanjoo Z, Haghjoo M (2018). New insights into predictors of cardiac implantable electronic device infection.. Tex Heart Inst J..

[38] Romeyer-Bouchard C, Da Costa A, Dauphinot V (2010). Prevalence and risk factors related to infections of cardiac resynchronization therapy devices.. Eur Heart J..

[39] Kleemann T, Becker T, Strauss M (2010). Prevalence of bacterial colonization of generator pockets in implantable cardioverter defibrillator patients without signs of infection undergoing generator replacement or lead revision.. Europace..

[40] Al-Khatib SM, Lucas FL, Jollis JG, Malenka DJ, Wennberg DE (2005). The relation between patients’ outcomes and the volume of cardioverter-defibrillator implantation procedures performed by physicians treating Medicare beneficiaries.. J Am Coll Cardiol..

[41] Chauhan A, Grace AA, Newell SA (1994). Early complications after dual chamber versus single chamber pacemaker implantation.. Pacing Clin Electrophysiol..

[42] Mittal S, Shaw RE, Michel K (2014). Cardiac implantable electronic device infections: incidence, risk factors, and the effect of the AigisRx antibacterial envelope.. Heart Rhythm..

[43] Mela T, McGovern BA, Garan H (2001). Long-term infection rates associated with the pectoral versus abdominal approach to cardioverter-defibrillator implants.. Am J Cardiol..

[44] Gil P, Fernandez Guerrero ML, Bayona JF (2006). Infections of implantable cardioverter-defibrillators: frequency, predisposing factors and clinical significance.. Clin Microbiol Infect..

[45] Brouwer TF, Yilmaz D, Lindeboom R (2016). Long-term clinical outcomes of subcutaneous versus transvenous implantable defibrillator therapy.. J Am Coll Cardiol..

[46] Olde Nordkamp LR, Dabiri Abkenari L, Boersma LV (2012). The entirely subcutaneous implantable cardioverter-defibrillator: initial clinical experience in a large Dutch cohort.. J Am Coll Cardiol..

[47] Looser PM, Saleh L, Thomas G, Cheung JW (2017). Systemic infection due to subcutaneous implantable cardioverter-defibrillator implantation: importance of early recognition and treatment of device pocket-related complications.. Heart Rhythm Case Rep..

[48] Tarakji KG, Wazni OM, Harb S, Hsu A, Saliba W, Wilkoff BL (2014). Risk factors for 1-year mortality among patients with cardiac implantable electronic device infection undergoing transvenous lead extraction: the impact of the infection type and the presence of vegetation on survival.. Europace..

[49] Olde Nordkamp LR, Knops RE, Bardy GH (2012). Rationale and design of the PRAETORIAN trial: a Prospective, RAndomizEd comparison of subcuTaneOus and tRansvenous ImplANtable cardioverter-defibrillator therapy.. Am Heart J..

[50] Tjong FV, Reddy VY (2017). Permanent leadless cardiac pacemaker therapy: a comprehensive review.. Circulation..

[51] Mangram AJ, Horan TC, Pearson ML, Silver LC, Jarvis WR (1999). Guideline for prevention of surgical site infection, 1999. Hospital Infection Control Practices Advisory Committee.. Infect Control Hosp Epidemiol..

[52] Seropian R, Reynolds BM (1971). Wound infections after preoperative depilatory versus razor preparation.. Am J Surg..

[53] Darouiche RO, Wall MJ,  Itani KM (2010). Chlorhexidine-alcohol versus povidone-iodine for surgical-site antisepsis.. N Engl J Med..

[54] Qintar M, Zardkoohi O, Hammadah M (2015). The impact of changing antiseptic skin preparation agent used for cardiac implantable electronic device (CIED) procedures on the risk of infection.. Pacing Clin Electrophysiol..

[55] Sastry S, Rahman R, Yassin MH (2015). Cardiac implantable electronic device infection: from an infection prevention perspective.. Adv Prev Med..

[56] Baddour LM, Epstein AE, Erickson CC (2010). Update on cardiovascular implantable electronic device infections and their management: a scientific statement from the American Heart Association.. Circulation..

[57] Reynbakh O, Akhrass P, Souvaliotis N (2018). Use of MPH hemostatic powder for electrophysiology device implantation reduces postoperative rates of pocket hematoma and infection.. Curr Med Res Opin..

[58] Chia PL, Foo D (2013). Use of oxidized regenerated cellulose to prevent pocket hematomas after cardiac electronic device implantation in patients on anticoagulants or dual antiplatelet therapy.. Int J Cardiol..

[59] Beton O, Saricam E, Kaya H (2016). Bleeding complications during cardiac electronic device implantation in patients receiving antithrombotic therapy: is there any value of local tranexamic acid?. BMC Cardiovasc Disord..

[60] Ohlow MA, Lauer B, Buchter B, Schreiber M, Geller JC (2012). Pocket related complications in 163 patients receiving anticoagulation or dual antiplatelet therapy: D-Stat Hemostat versus standard of care.. Int J Cardiol..

[61] Birnie DH, Healey JS, Wells GA (2013). Pacemaker or defibrillator surgery without interruption of anticoagulation.. N Engl J Med..

[62] Birnie DH, Healey JS, Wells GA (2018). Continued vs. interrupted direct oral anticoagulants at the time of device surgery, in patients with moderate to high risk of arterial thrombo-embolic events (BRUISE CONTROL-2).. Eur Heart J..

[63] Bratzler DW, Houck PM, Surgical Infection Prevention Guidelines Writers Workgroup (2004). Antimicrobial prophylaxis for surgery: an advisory statement from the National Surgical Infection Prevention Project.. Clin Infect Dis..

[64] Kusumoto FM, Schoenfeld MH, Wilkoff BL (2017). 2017 HRS expert consensus statement on cardiovascular implantable electronic device lead management and extraction.. Heart Rhythm..

[65] Krahn AD, Longtin Y, Philippon F (2018). Prevention of arrhythmia device infection trial: the PADIT trial.. J Am Coll Cardiol..

[66] Tarakji KG, Mittal S, Kennergren C (2019). Antibacterial envelope to prevent cardiac implantable device infection.. N Engl J Med..

